# Characterization of the Ruminal Microbiome of Water Buffaloes *(Bubalus bubalis)* Kept in Different Ecosystems in the Eastern Amazon

**DOI:** 10.3390/ani13243858

**Published:** 2023-12-15

**Authors:** Gerlane Nunes Noronha, Melanie K. Hess, Ken G. Dodds, André Guimarães Maciel e Silva, Shirley Motta de Souza, Jamile Andréa Rodrigues da Silva, Diego Assis das Graças, Thomaz Cyro Guimarães de Carvalho Rodrigues, Welligton Conceição da Silva, Éder Bruno Rebelo da Silva, Peter H. Janssen, Hannah M. Henry, Suzanne J. Rowe, Vinicius Costa Gomes de Castro, José de Brito Lourenço-Júnior

**Affiliations:** 1 Postgraduate Program in Animal Science (PPGCAN), Institute of Veterinary Medicine, Brazilian Agricultural Research Corporation (EMBRAPA), Castanhal 68746-360, Brazil; gerlanenoronha@yahoo.com.br (G.N.N.); andregms@ufpa.br (A.G.M.e.S.); thomazguimaraes@yahoo.com.br (T.C.G.d.C.R.); eder.b.rebelo@gmail.com (É.B.R.d.S.); vinicius.c.gomes@hotmail.com (V.C.G.d.C.); joselourencojr@yahoo.com.br (J.d.B.L.-J.); 2 Invermay Agriculture Centre, AgResearch, Mosgiel 9053, New Zealand; melanie.k.hess@gmail.com (M.K.H.); ken.dodds@agresearch.co.nz (K.G.D.); hannah.henry@agresearch.co.nz (H.M.H.); suzanne.rowe@agresearch.co.nz (S.J.R.); 3 Federal Institute of Education, Science and Technology, South of Minas Gerais, Pouso Alegre 37550-000, Brazil; motta.shirley@hotmail.com; 4 Institute of Animal Health and Production, Federal Rural University of the Amazônia (UFRA), Belem 66077-830, Brazil; jamileandrea@yahoo.com.br; 5 Laboratory of Biological Engineering, Guamá Science and Technology Park, Belem 66075-750, Brazil; diegoassis@ufpa.br; 6 Grasslands Research Centre, AgResearch, Palmerston North 4410, New Zealand; peter.janssen@agresearch.co.nz

**Keywords:** Amazonia, ruminal microorganisms, restriction enzyme-reduced representation sequencing

## Abstract

**Simple Summary:**

Maximizing ruminant production relies on enhancing rumen fermentation efficiency, necessitating an understanding of the ruminal microbiome and its environmental impacts. This study aimed to characterize buffalo microbiomes in the eastern Amazon across seasons and ecosystems. The study included three grazing systems: Baixo Amazonas (BA), Continente do Pará (CP), Ilha do Marajó (IM), and a confinement system: Tomé-Açu (TA). Seventy-one samples from male crossbred buffaloes were analyzed for ruminal microbial community structure, along with the compositions of their diets. Bacterial and Archaeal taxa were identified, with 61 genera recognized. Taxonomic composition similarities were observed across ecosystems. Twenty-three bacterial genera significantly differed between the confinement and other ecosystems. Among Archaea, abundances of *Methanomicrobium* and *Methanosarcina* varied between ecosystems. Diet (available or offered) exerted the most influence on the ruminal microbiota. This research provides insights into buffalo microbiomes in the Amazon, vital for optimizing ruminant production sustainably.

**Abstract:**

Increasing the efficiency of rumen fermentation is one of the main ways to maximize the production of ruminants. It is therefore important to understand the ruminal microbiome, as well as environmental influences on that community. However, there are no studies that describe the ruminal microbiota in buffaloes in the Amazon. The objective of this study was to characterize the rumen microbiome of the water buffalo (*Bubalus bubalis*) in the eastern Amazon in the dry and rainy seasons in three grazing ecosystems: Baixo Amazonas (BA), Continente do Pará (CP), Ilha do Marajó (IM), and in a confinement system: Tomé-Açu (TA). Seventy-one crossbred male buffaloes (Murrah × Mediterranean) were used, aged between 24 and 36 months, with an average weight of 432 kg in the rainy season and 409 kg in the dry season, and fed on native or cultivated pastures. In the confinement system, the feed consisted of sorghum silage, soybean meal, wet sorghum premix, and commercial feed. Samples of the diet from each ecosystem were collected for bromatological analysis. The collections of ruminal content were carried out in slaughterhouses, with the rumen completely emptied and homogenized, the solid and liquid fractions separated, and the ruminal pH measured. DNA was extracted from the rumen samples, then sequenced using Restriction Enzyme Reduced Representation Sequencing. The taxonomic composition was largely similar between ecosystems. All 61 genera in the reference database were recognized, including members of the domains Bacteria and Archaea. The abundance of 23 bacterial genera differed significantly (*p* < 0.01) between the Tomé-Açu confinement and other ecosystems. *Bacillus*, *Ruminococcus*, and *Bacteroides* had lower abundance in samples from the Tomé-Açu system. Among the Archaea, the genus *Methanomicrobium* was less abundant in Tomé-Açu, while *Methanosarcina* was more abundant. There was a difference caused by all evaluated factors, but the diet (available or offered) was what most influenced the ruminal microbiota.

## 1. Introduction

Currently, the buffalo population in Brazil is around 1.6 million head, mostly in the northern region of the country [1]. The eastern Amazon holds 67% of this population and the largest amount of buffalo farming for milk and meat is in the state of Pará. Buffaloes arrived in Brazil in 1890, originally from India, and they adapted well to Brazil’s tropical regions because of the similarity to their original environment [2].

In flooded or very dry areas of the Amazon, buffaloes perform better than cattle. It is believed that this advantage is due to their digestive performance, as they display greater degradation of the fibrous fraction and greater retention of nitrogen, especially on low-protein diets [3]. Ruminal fermentation of plant material allows ruminants to degrade the lignocellulose present in plants and convert non-protein nitrogen into microbial protein. This occurs through the action of the ruminal microbiota, and one of the main factors contributing to the digestion of the ingested substrate is the nature of the diet [4]. The final products of fermentation are short-chain fatty acids which are absorbed by the rumen and used as an energy source for the host animal’s maintenance and growth [5].

A great diversity of microorganisms, such as bacteria, archaea, protozoa, fungi, and viruses, make up the ruminal ecosystem. However, bacteria are the largest and most important group, due to their greater participation in the degradation of fibers and protein sources, ten times more efficient than other microbes found in the rumen [6]. Studies on the classification of this microbiota are relatively recent, but the advancement of applied technologies, such as gene sequencing and metagenomic analysis, has allowed greater knowledge and characterization of the ruminal ecosystem [7].

Genetic sequencing makes it possible to identify portions of genes or the entire genome, which facilitated mapping and genetic cloning, in addition to contributing to the beginning of biodiversity studies [8]. Next-generation sequencing (NGS) has provided numerous benefits over previous methods, such as being faster, yielding more data, not requiring DNA cloning, and reducing costs [9]. One way to reduce genome complexity is Restriction Enzyme Reduced Representation Sequencing (RE-RRS) [10,11]. This process involves restriction enzyme digestion of genomic DNA (for example, by PstI with cut site CTGCA | G), followed by a size selection step and fragment sequencing [10]. The specific fraction to be sequenced is able to capture useful information about microbial composition and diversity at a reduced cost compared to metagenomic sequencing when applied to animal selection [10,11].

The study of the ruminal microbiome is still in its early stages in Brazil, and there has been no research to characterize the microbial communities in the rumen of buffaloes. Most studies have focused on feed efficiency with different diets. The hypothesis in this study was that there is a difference in the ruminal microbiota of buffaloes raised in different ecosystems in the eastern Amazon. Knowledge of environmental variability is important if RE-RRS is to be used for selection purposes, to determine improved feed utilization or reduced environmental impacts. Our objective was to identify the ruminal microbiome of buffaloes in three different grazing ecosystems (Baixo Amazonas (BA), Continente do Pará (CP), Ilha do Marajó (IM) and in feedlots at Tomé-Açu (TA) and compare these microbial profiles between locations, seasons (dry or rainy), and ruminal content fraction (solid or liquid).

## 2. Materials and Methods

### 2.1. Ethical Aspects

The experimental procedures used in this study were approved by the Animal Ethics Committee of the Federal Rural University of the Amazon (CEUA/UFRA N° 4542190820).

### 2.2. Ecosystems

In this study, RE-RRS microbial profiles were generated from 138 samples of rumen contents from buffaloes kept in natural grazing environments or a confinement system in the eastern Amazon. We considered four diverse ecosystems in our study, as described below. The geographic locations of the collection sites are described in Table 1.

#### 2.2.1. Ilha do Marajó (IM)

The IM location is a rural farm on the Ilha do Marajó (Island of Marajó) with native pastures of dryland/wetland in the municipality of Soure, Pará. Its climate is rainy tropical, and the period of greatest precipitation occurs between January and June, when the intensity of the rains is so great that an extensive part of the island is flooded. The driest period is between September and November [12]. The livestock system is extensive, traditionally in open fields. Grasses native to these flooded areas are *Panicum elephantipes*, *Leersia hexandra*, and *Hymenachne amplexicaulis*, where few pastures are formed.

#### 2.2.2. Baixo Amazonas (BA)

Th BA location is a farm in Santarém, a municipality in the of Baixo Amazonas Mesoregion. Its climate is hot and humid, typical of tropical forests. The months of December to June are the rainiest, while July to November are the least rainy [12]. The farming system is extensive, with native pastures typical of flooded lands. Some of the grasses grown in the pasture are *Panicum maximum* cv. Mombaça and *Brachiaria brizantha*.

#### 2.2.3. Continente do Pará (CP)

The CP location is a rural property in the municipality of Nova Timboteua, in the Mesoregion of Northeast of Para. The climate is of the Am type, according to the Köppen classification [12], with average annual temperatures of 26.1 ℃, 85% relative humidity, and 2250 mm of precipitation, distributed over two periods: rainier from January to June and less rainy from July to December. Currently, the vegetation cover is formed by secondary forest, agricultural areas, and pastures, caused by the human modification of the native environment [13]. The breeding system is characterized by cultivated grassland, with grasses such as Panicum maximum cv. Mombaça, *Brachiaria humidicola*, and *Urochloa* syn. *Brachiaria*.

#### 2.2.4. Tomé-Açu (TA)

The confinement system studied is on a property of an animal exporting company, located in the municipality of Tomé-Açu, Pará, also in the mesoregion northeast of Para. The climate type is Am, according to the Köppen classification, hot and humid, with the highest rainfall, about 150 mm/month, occurring from December to May [12,14]. In this breeding system, the animals were fed with sorghum silage, soybean meal, wet sorghum pre-mix, and commercial feed.

### 2.3. Pasture Sampling and Analysis

Samples of pasture in the three grazing systems were collected using the square method, with a PVC plastic frame (polyvinyl chloride) enclosing an area of 1.0 m^2^ [15]. In CP, where there was a rotational grazing system, the collection was carried out at the exit height (40 cm) of the animals in the paddock, collecting the upper part of the plant, knowing that the entry height was 60 cm. In the IM and CP systems, pasture collection was carried out close to the ground. The samples were quartered and four 1 kg subsamples were transported in transparent plastic bags in a cooler with ice until the time of analysis. In addition, samples of feed offered to animals in confinement (TA) were also collected.

Bromatological analyses of the diets were performed at the Animal Nutrition Laboratory at UFPA, Castanhal campus, Pará, Brazil. The feeds were analyzed for dry matter (DM; method INCTCA N-00/1), ash (method INCT-CA M-001/1), crude protein (CP; method INCT-CA N-001/1), ether extract (EE; method INCT-CA G005/1), neutral detergent fiber corrected for ash and protein (NDFap; methods INCT-CA F-002/1, INCT-CA M-002/1, and INCT-CA N-004/1), acid detergent fiber corrected for ash and protein (ADFap; methods INCT-CA F-004/1, INCT-CA M-003/1, and INCT-CA N-005/1), and acid detergent lignin (ADL; method INCT-CA F-005/01), using the methods recommended by the National Institute of Science and Technology in Animal Science (INCT-CA) [16]. Total digestible nutrients (TDN) were calculated according to the Clemson University equation: TDN = 93.59 − (ADF × 0.936).

### 2.4. Animals

Sixty rumen samples were collected from 60 male buffaloes, Murrah × Mediterranean crossbreeds, aged between 24–36 months, in the BA, PA, and IM systems across two seasons (dry and wet season). A sample was collected from each of 11 male buffaloes, aged 18 months, in the confinement system (TA). The samples from the confinement system were collected only in the rainy season, since there was no variation in the diet with the season. There were 10 animals in each ecosystem and period, except in the confinement system where there were 11. The average ages and weights of the animals at the point of rumen sample collection within each ecosystem and period are shown in Table 2. The average weight of the animals was 432 kg at the end of the rainy season and 409 kg at the end of the dry season.

### 2.5. Sampling of Rumen Contents and DNA Extraction

The animals were slaughtered after 18 h of fasting and rumen samples were immediately collected, in regulated commercial slaughterhouses, in accordance with the Brazilian federal legislation in force, Decree 9013 of 29 March 2017. The rumen of each animal was emptied, the total rumen content was homogenized and separated by a tissue bag into solid and liquid fractions, 25 g and 25 mL, respectively, and immediately transferred to 50 mL Falcon tubes with 25 mL of buffer solution (EB; 100 mM Tris, 10 mM ethylenediaminetetraacetic acid (EDTA), 0.15 M NaCl, pH 8.0 with HCl), transported on ice and stored at −80 °C [17]. This resulted in 2 samples per animal (the solid and liquid fractions), and 142 samples in total. The pH of the ruminal liquid was measured immediately after separation from the solid fraction using a pH meter (model pH Pro-02-0519, AKSO, São Leopoldo, Rio Grande do Sul, Brazil).

DNA was extracted from the samples in the molecular biology laboratory of the Federal University of Pará-UFPA, Brazil, using a commercial kit (FastDNA Spin Kit for Soil; MP Biomedicals, Irvine, CA, USA), following the instructions provided by the manufacturer.

### 2.6. Sequencing

All 142 samples were sent, on dry ice, from Pará, Brazil, to New Zealand, where they were sequenced in the laboratory of AgResearch Ltd. at Invermay. Restriction Enzyme Reduced Representation Sequencing (RE-RRS) was used to sequence rumen samples, as described by Hess et al. [11]. Briefly, 100 ng of DNA was normalized to 20 ng/µL using PicoGreen and digested by the enzyme PstI (CTGCA|G). After digestion, the barcodes and adapters were attached to the samples [18] and fragments between 193 and 318 bp were selected using Pippin Prep (SAGE Science, Beverly, MA, USA), and then sequenced. The library was purified (QIAquick 96 PCR Purification Kit; Qiagen, Hilden, Germany) and the eluted DNA was amplified by PCR using primers and conditions described by Elshire et al. [18]. The library was sequenced in a single run of Illumina HiSeq2500, which generates reads of up to 101 bp of each fragment, including adapters, the barcode sequence and the DNA of interest.

### 2.7. Bioinformatics Processing

The sequenced reads were demultiplexed using GBSX and cut using Cutadapt [19] to remove sequences shorter than 40 base pairs. On average, 1,572,072 usable reads were obtained per sample. Samples with less than 100,000 reads, after demultiplexing and cutting, were considered “failed” and were removed from further analysis.

We used the reference-based pipeline described by Hess et al. [10,11], which compares the sequenced reads to bacterial and archaeal genomes from the Hungate 1000 Collection [20], with the addition of four *Quinella* genomes [21]. The microbial profile contained reads assigned to one of the 61 genera in this reference database. Two other microbial profiles were generated: (1) A Proportion matrix, in which the number of reads attributed to each genus was divided by the total number of reads attributed to the reference database for that sample; and (2) A Log10 Proportions Matrix, in which one was added to the counts for each genus and this was divided by the total number of reads attributed to each genus plus the number of genera, before that number was logged (base 10).

Ruminal bacterial diversity was estimated through diversity and richness indices, using the phyloseq [22], vegan [23] and ggplot [24] packages in R Studio software v.1.0.136 [25].

### 2.8. Statistical Analysis

Statistical analyses were performed with the aid of R/RStudio and the packages lme4, according to Bates et al. [26], predictmeans [27] and lmerTest [28]. The log10 proportion of each genus was analyzed using the model:

*y = LocType + LocType:Local + LocType:Season + Fraction + Animal + e*

where *y* is the log10 proportion for each genus, *e* is the residual, *Animal* is the animal ID set as a random effect, *Loctype* tests the difference between free (BA, IM, and CP) and confinement systems (TA), *LocType:Local* tests the difference between free locations, *LocType:Season* tests the difference between dry season or rainy season, and *Fraction* tests the difference between the solid and liquid fractions of the rumen content of the samples. LS means, standard errors, and F tests were obtained using the predictmeans package, with α = 0.001 for the significance test.

## 3. Results

Table 3 shows the chemical composition of the diets consumed by animals from each ecosystem in each season. The mean pH values of rumen samples collected from the buffaloes in the three grazing ecosystems were between 7.18 and 7.53, while in the confinement system it was 6.86 (Table 3).

High-throughput microbial profiling of buffalo rumen samples using RE-RRS resulted in 16 ± 2.8% of reads assigned to the reference database at the genus level. This is a little lower than the 23.4 ± 3.7% reads attributed to the reference database using the same restriction enzyme applied to sheep rumen content samples by Hess et al. [10].

**Figure 1:**
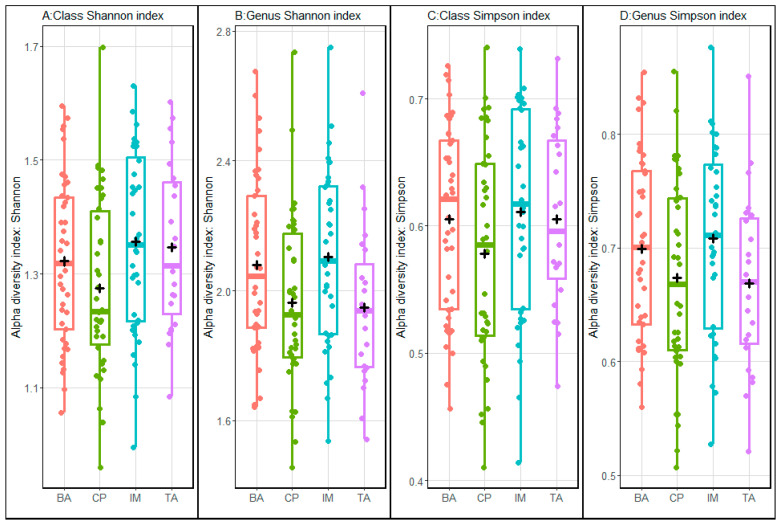
Shannon (panels **A** and **B**) and Simpson (panels **C** and **D**) microbial alpha diversity indices comparing different ecosystems (BA—Baixo Amazonas, CP—Continente do Pará, IM—Ilha do Marajó, TA—Tomé-Açu). The analyses were performed using microbial composition data classified at the class (panels **A** and **C**) and genus (panels **B** and **D**) level.

**Figure 2:**
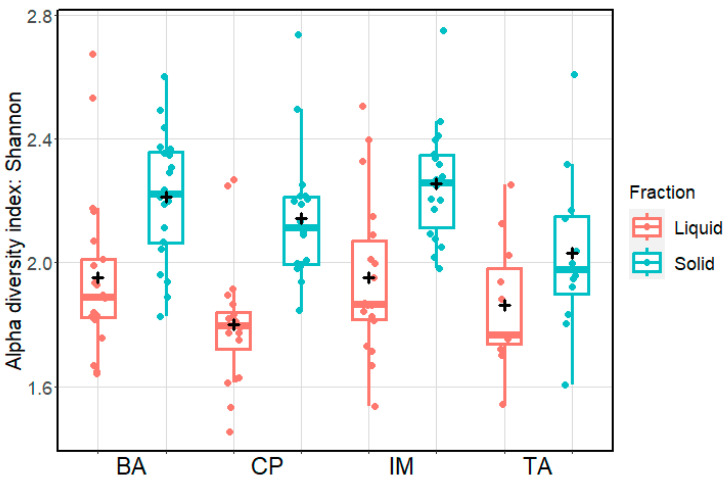
Shannon microbial alpha diversity index using microbial composition data at the genus level and comparing the ecosystems (BA—Baixo Amazonas, CP—Continente do Pará, IM—Ilha do Marajó, TA—Tomé-Açu) and the liquid and solid fractions of rumen contents.

**Figure 3:**
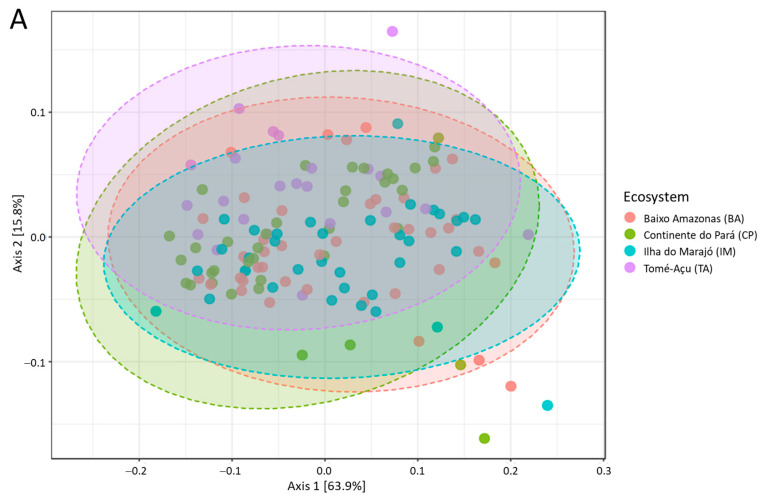

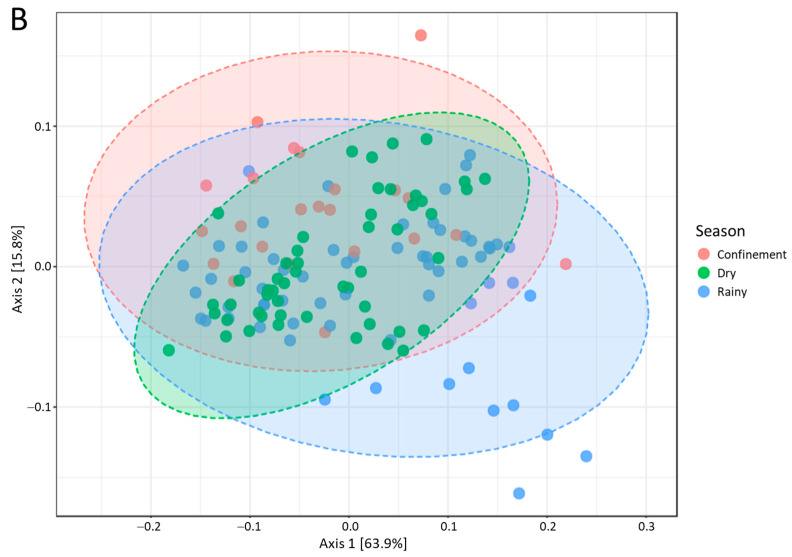
Measure of microbial beta diversity at the genus level: (**A**) between ecosystems; (**B**) between seasons (dry and rainy) and the confinement system.

Alpha and beta diversity indices were used to compare the microbial communities. Shannon and Simpson alpha diversity indices (Figure 1) indicated a greater level of diversity in the IM ecosystem when compared to the other ecosystems. The solid fraction contained greater diversity than the liquid fraction (Figure 2). Comparisons of beta diversity suggested there was little variation in microbial species composition between ecosystems (Figure 3A) and between the seasons of the year (Figure 3B).

**Figure 4:**
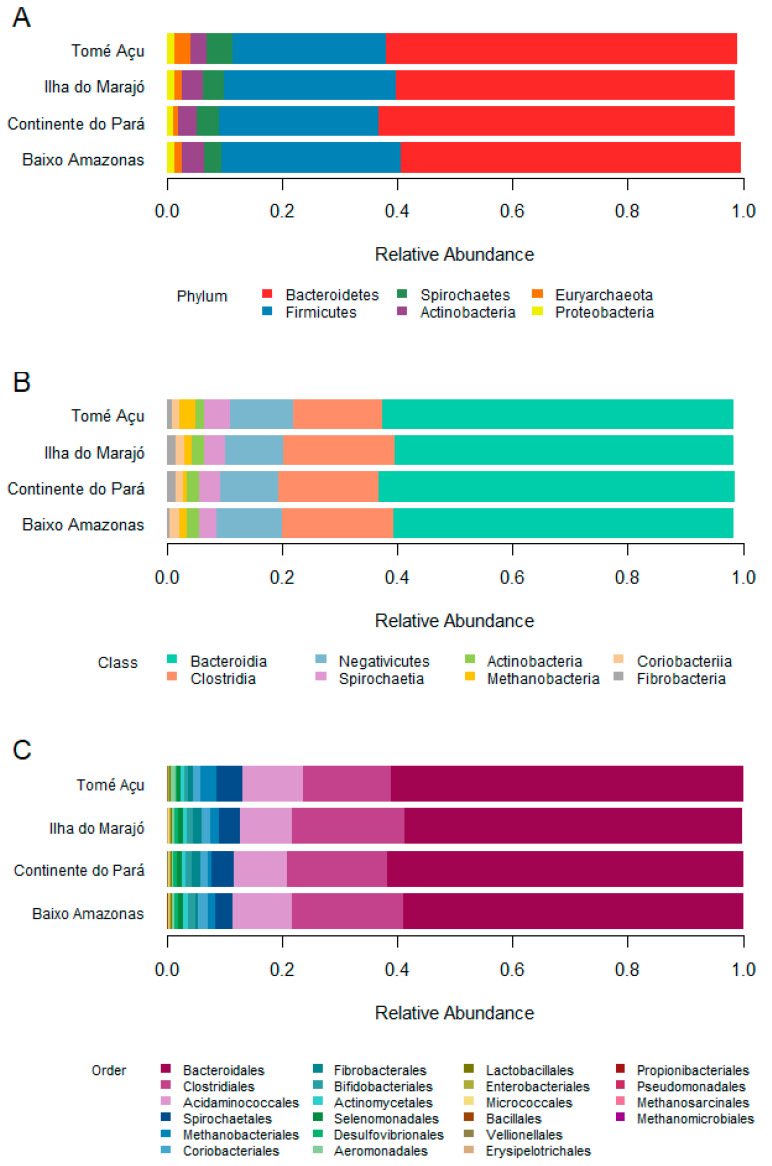
Taxonomic classification at the level of (**A**) phylum, (**B**) class, and (**C**) order of rumen microbial communities from the four ecosystems.

The bacteria and archaea were classified at different taxonomic ranks: phylum, class, order, family, and genus. The taxonomic composition was not very variable between the BA, CP, IM, and TA ecosystems. The Bacteroidetes and Firmicutes were the predominant bacterial phyla, and among the archaea, the phylum Euryarchaeota was identified (Figure 4A). At the class level, the taxonomic profile revealed the predominance of three classes: Bacteroidia, Clostridia, and Negativicutes. The classes of Archaea present were Methanomicrobia and Methanobacteria (Figure 4B). The most abundant bacterial orders were Bacteroidales, Clostridiales, and Acidaminococcales. The predominant order of Archaea was Methanobacteriales (Figure 4C).

**Figure 5:**
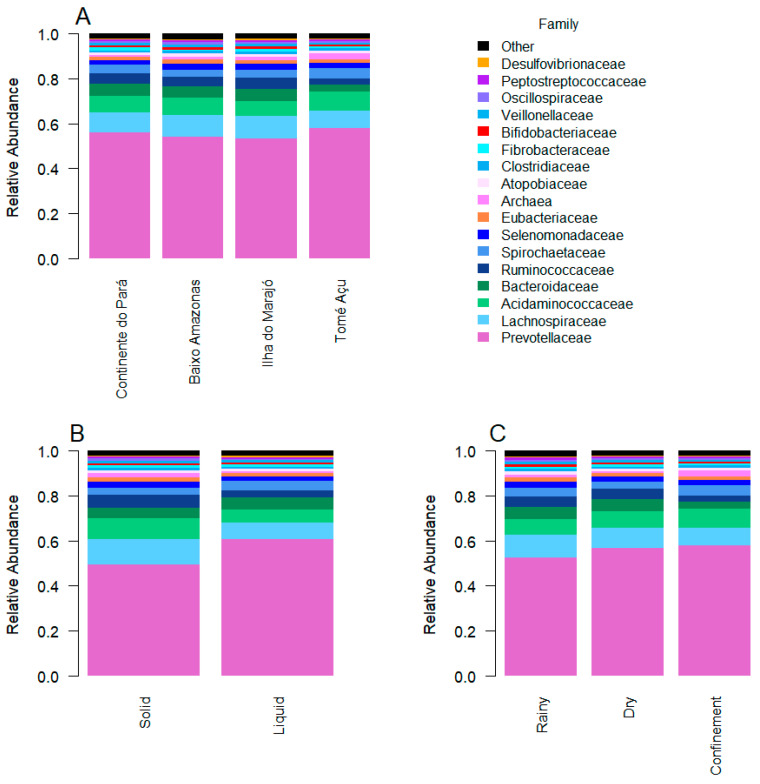
The taxonomic composition and relative abundance at the family level for ruminal microbiome. (**A**) Ecosystem, (**B**) fraction, and (**C**) season. Note that Archaea were classified together for this analysis.

The 61 genera in our reference database represent 38 families, 3 of which are Archaea. In all ecosystems, seasons, and fractions, the families *Prevotellaceae*, *Lachnospiraceae*, and *Acidaminococcaceae* were the most abundant (Figure 5). The biggest driver of differences in microbial profiles was whether the sample was from the solid or the liquid fraction. Of the most abundant families, *Prevotellaceae* and *Bacteroidaceae* were more abundant in the liquid fraction than the solid fraction, while *Lachnospiraceae* and *Acidaminococcaceae* were more abundant in the solid fraction than the liquid fraction (Figure 5B). The microbial profiles from the TA ecosystem were the least similar to the other ecosystems (Figure 5A), and these differences were largely driven by differences in abundance of *Ruminococcaeae* (lower in TA), *Bacteroidaceae* (lower in TA), *Spirochaetaceae* (higher in TA), and *Succinivibrionaceae* (higher in TA) and Archaea (higher in TA). *Prevotellaceae* and *Acidaminococcaceae* were less abundant in the rainy season than in the dry season, while *Lachnospiraceae* was more abundant (Figure 5C). Three families of archaea were identified: *Methanobacteriaceae*, *Methanomicrobiaceae*, and *Methanosarcinaceae*. The confinement system showed the highest abundance of Archaea (Figure 5C), and among the fractions, the solid fraction had a greater predominance of archaea than the liquid fraction (Figure 5B).

**Figure 6:**
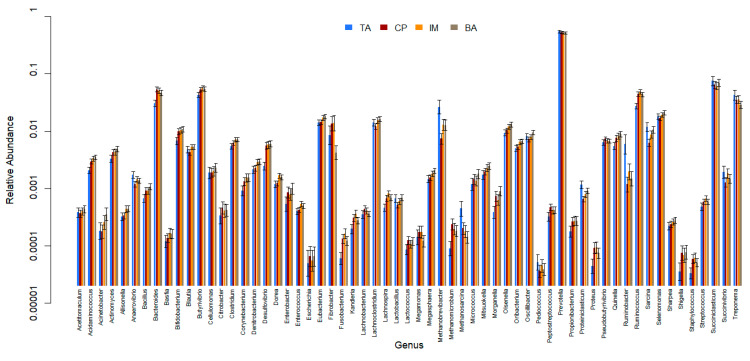
Relative abundance of bacterial and archaeal genera among ecosystems (BA—Baixo Amazonas, CP—Continente do Pará, IM—Ilha do Marajó, TA—Tomé-Açu). Error bars show 95% confidence intervals.

**Figure 7:**
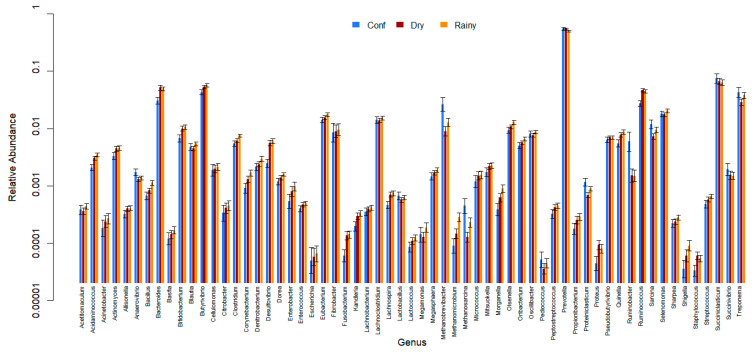
Relative abundance of bacterial and archaeal genera from the confinement system (Conf) and seasons (Dry and Rainy). Error bars show 95% confidence intervals.

**Figure 8:**
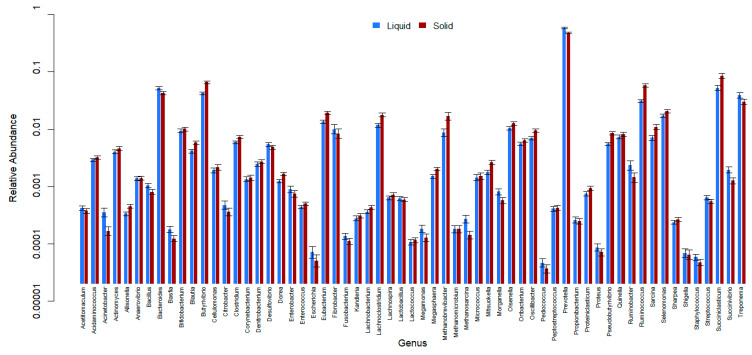
Relative abundance of bacterial and archaeal genera in solid and liquid fractions of buffalo rumen content. Error bars show 95% confidence intervals.

**Figure 9:**
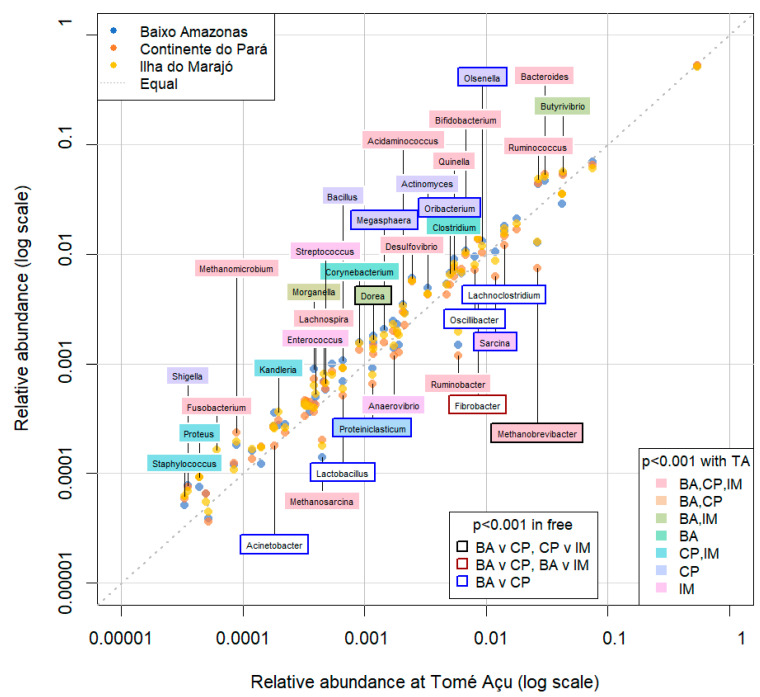
Genus level composition of the microbial communities of buffalo rumen at the four different locations (BA—Baixo Amazonas, CP—Continente do Pará, IM—Ilha do Marajó, TA—Tomé-Açu). The three gazing systems (BA, CP, IM) are compared to the confinement system (TA).

**Figure 10:**
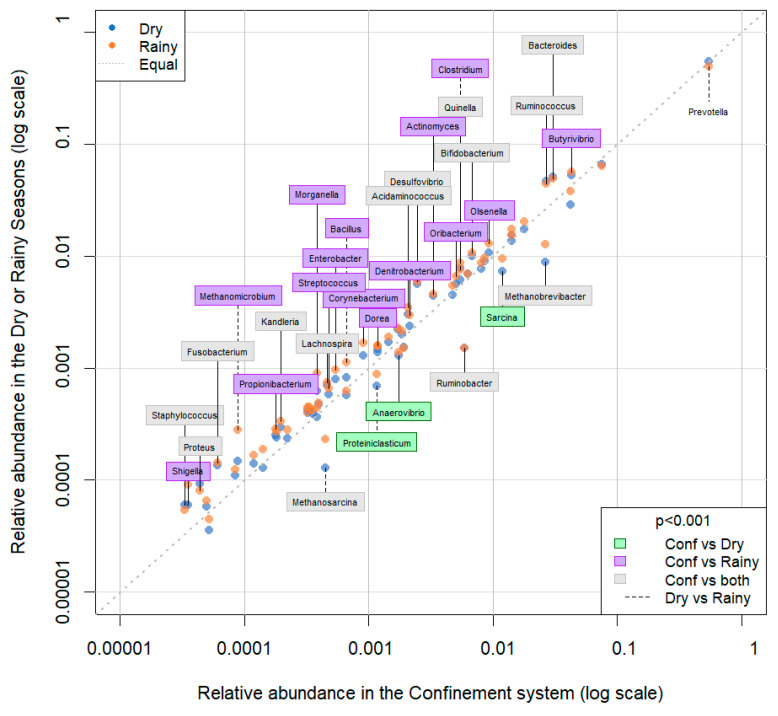
Genus level composition of the microbial communities of buffalo rumen in the dry and rainy seasons in the grazing systems compared to the confinement system.

**Figure 11:**
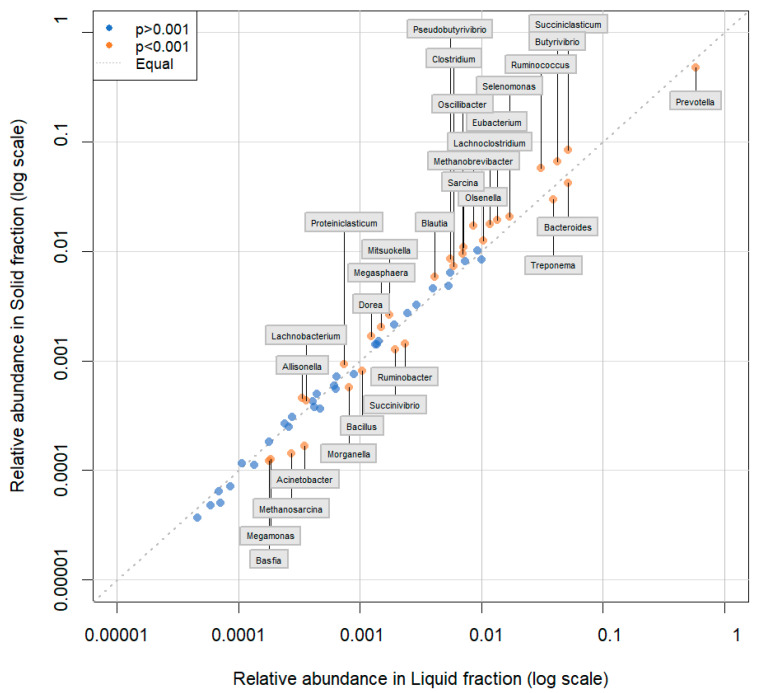
Average relative abundance of the microbial community of ruminal buffalo, determined by pipeline (solid and liquid fractions).

All 61 genera in the reference database were identified in samples from all of the ecosystems (CP, BA, IM, and TA). *Prevotella* was the most abundant genus, followed by *Succiniclasticum*, *Bacteroides*, *Butyrivibrio*, and *Ruminococcus* (Figure 6, Figure 7 and Figure 8 and Table S1). There were 25 genera that differed significantly (*p* < 0.001) in relative abundance between the confinement and free systems, 23 of which were bacterial and two archaeal (Figure 9). Seven genera differed significantly between locations with the free system (*Dorea*, *Fibrobacter*, *Lachnoclostridium*, *Oscillibacter*, *Proteiniclasticum*, *Sarcina*, and the archaeal genus *Methanobrevibacter*) (Figure 9). When comparing the seasons and the confinement system, six genera differed significantly, four bacterial (*Bacillus*, *Clostridum*, *Prevotella*, and *Proteiniclasticum*) and two archaeal (*Methanomicrobium* and *Methanosarcina*) (Figure 10). There was a greater number of reads per sample in the solid fraction (mean 1,577,199) than in the liquid fraction (mean 1,567,111). Thirty genera were observed to be significantly different (*p* < 0.001) between the fractions (liquid and solid); twenty-eight bacterial and two archaeal (Figure 11).

## 4. Discussion

The ruminal microbiota may show some difference between ruminant species, but a major factor influencing the microbial composition in the rumen is the animal’s diet [29,30,31,32]. Many studies have focused on different aspects of the ruminal microbiota, such as composition, function, and how efficiently the animal digests feed. This study aimed to characterize the ruminal microbial community of buffaloes in natural habitats and in a confinement system in the eastern Amazon, through sequencing using a reference based RE-RRS pipeline [10,11].

Microbial diversity indices were higher in the solid fraction than in the liquid fraction, in all ecosystems, probably because this fraction contains the greatest range of structural and chemical diversity and microbes adhere to the ingested feed to initiate degradation on the many complex components of their feed. This result agrees with the findings of AlZahal et al. [31], who observed that bacterial diversity changes according to the diet and location of the rumen content sample. Reported microbial diversity is also limited by the reference database.

The results show that *Prevotellaceae* and *Bacteroidaceae* are the main families of the phylum Bacteroidetes present in the buffalo rumen in all studied ecosystems, while the *Lachnospiraceae*, *Acidaminococcaceae*, and *Ruminococcaceae* families were prevalent in the phylum Firmicutes, in agreement with previous studies by AlZahal et al. [31] and Nathani et al. [32], who observed similar results in cows and Jaffrabadi buffalo, respectively.

The present research demonstrated that the genera *Prevotella*, *Succiniclasticum*, *Butyrivibrio*, and *Bacteroides* were predominant among ecosystems. Henderson et al. [30] found that the genera *Prevotella* and *Butyrivibrio* were among the most abundant found in rumen content of different species of ruminants around the world. *Prevotella* was the most abundant microorganism among the ruminal bacterial genera in the samples described here and represented almost 56% of the identified taxa (Figure 6). *Prevotella* and *Succiniclasticum* were predominant in the TA system, where the animals had a concentrate-based diet, rich in starch. *Prevotella*’s main function in the rumen is likely the degradation and use of starch, the degradation of polysaccharides such as xylans and pectins in the cell wall of plants, but it does not degrade cellulose [33,34,35]. *Succiniclasticum* ferments succinate to propionate and previous studies indicate that the abundance of this microorganism increases with diets rich in concentrate [36], which can explain the greater abundance of this genus in ruminal buffalo samples raised in confinement (TA).

A group of 25 taxa showed a significant difference (*p* < 0.001) in relative abundance between the TA feedlot ecosystem and the grazing ecosystems (Figure 9). Seven genera differed significantly between the grazing ecosystems. *Dorea* had a significantly greater abundance in the CP ecosystem in relation to BA and IM, but it did not differ from TA (Figure 9). *Butyrivibrio* had lower abundance in TA, compared to the BA and IM ecosystems. *Butyrivibrio* is a fibrolytic microbe, despite being able to use starch to produce butyrate [37], which explains the reduction in its abundance in the rumen of buffaloes raised in TA, as the diet was rich in concentrate and with lower NDF than in other ecosystems. *Bacteroides* were significantly less abundant in TA than in the other ecosystems, where the animals had diets rich in forage.

Archaea were more abundant in TA buffalo samples, compared to samples from the BA, IM, and CP ecosystems. The most abundant archaea were in the genus *Methanobrevibacter*, and similar patterns occurred in water buffaloes [38,39]. *Methanobrevibacter* had a significantly greater abundance in TA (*p* < 0.001) compared to the other locations.

There were six genera that differed significantly between the seasons (dry and rainy), two of which were archaea (*p* < 0.001). *Bacillus* and *Clostridium* had a significantly higher average abundance in the rainy season than in the dry season or the confined system (TA). *Proteiniclasticum* was more abundant in the rainy season than in the dry season. The rainy season presented pasture with a higher proportion of protein than the dry season or confinement system (Table 1). These genera belong to the phylum Firmicutes and degrade cellulose, and the results corroborate studies in buffaloes and cattle, which indicate that diets rich in fiber have a greater abundance of microorganisms belonging to this phylum [32,40]. The genus *Prevotella* was significantly more abundant in the dry than in the rainy season. *Prevotella* is a non-fibrous carbohydrate-fermenting member of *Bacteroidetes* [33], which may explain why it had a lower abundance when the feeds had a lower proportion of non-fibrous carbohydrate in the rainy period. Nathani et al. [32] showed that *Prevotella* decreases with increasing roughage in the buffalo diet.

Archaea were more abundant in the confinement system than in the grazing systems in the dry or rainy seasons. Among the identified archaea, *Methanomicrobium* had a significantly higher abundance (*p* < 0.001) in samples from the rainy period in relation to the dry season and confinement. *Methanosarcina* had a higher relative abundance in confinement than in the rainy season, which in turn had a higher relative abundance than in the dry season. Previous studies have described that increasing concentrated, digestible NDF in the diet can increase the abundance of archaea in the rumen content [41].

Genera of the phylum Firmicutes were observed with greater abundance in the solid fraction, when compared to the liquid fraction. In contrast, genera of the phylum Bacteroidetes were more abundant in the liquid fraction than in the solid fraction. Similar results have also been described previously in buffaloes and cattle [32,42], At the family level, *Prevoletaceae* and *Bacteroidaceae* were more abundant in the liquid fraction, while *Lachnospiraceae* and *Ruminococcaceae* were more abundant in the solid fraction, probably because they develop fibrolytic activities in the rumen and a large part of the fiber can be found in the solid fraction of the ruminal content [31].

There were 30 genera with a significant difference between fractions. Eighteen bacterial genera, including *Clostridium* and *Eubacterium*, and one archaeal genus had significantly higher abundance (*p* < 0.001) in the solid fraction (Figure 11), where most substrate degradation occurs. Similar results, with a greater abundance of *Clostridium* and *Eubacterium* in the solid fractions, have also been described in cattle and buffaloes [32,42].

In the liquid fraction, ten bacterial genera and one archaeal genus showed significantly greater abundance (*p* < 0.001) compared to the solid fraction. The genus *Prevotella* was the most abundant in both fractions, but it had a higher relative abundance in the liquid fraction, in agreement with previous research in lactating cows and buffalo fed on roughage [32,43]. *Prevotella* is a polysaccharide degrader [33], and the liquid fraction of the rumen content contains dissolved sugars that can be metabolized without the need for fibrolytic attack [44]. Amylolytic genera also had greater relative abundance in the liquid fraction, such as *Bacteroides*, *Succinivibrio*, and *Ruminobacter. Methanosarcina* was the archaea with a higher relative abundance (*p* < 0.001) in the liquid fraction compared to the solid fraction, although its total abundance was low. *Methanobrevibacter* had a greater relative abundance in the solid fraction compared to the liquid fraction.

## 5. Conclusions

The identification allowed the capture of all 61 genera tested from the content of the rumen of buffaloes among the studied ecosystems. with *Prevotella*, *Succiniclasticum*, *Bacteroides*, *Butyrivibrio*, and *Ruminococcus* being the most prevalent genera of microorganisms. The rumen microbiome of buffalo varies with ecosystem and season, but these differences are small.

## Figures and Tables

**Table 1:** Location and climate information for the ecosystems in our study.

Ecosystem	Lattitude	Longitude	Altitude (m)	Köppen Classification	Rainfall (mm)	Av. Temp. (°C)	Av Humidity (%)
IM	0°39′27.89″ S	48°42′35.01″ W	7	Am	2500	27	85
BA	02°41′48.83″ S	54°38′35.43″ W	108	Am	2000	26	86
CP	01°12′52.63″ S	47°24′30.94″ W	53	Am	2467	26	86
TA	02°17′28.32″ S	48°05′56.11″ W		Am			

**Table 2:** Age and weight of buffaloes at the time of rumen sample collection in rainy or dry seasons.

Ecosystem	Weight (kg)		Age (Months)	
	Rainy	Dry	Rainy	Dry
Ilha do Marajó (IM)	396	418	24	36
Baixo Amazonas (BA)	445	454	24	36
Continente do Pará (CP)	212	432	24	36
Tomé-Açu (TA)	433		18	

**Table 3:** Proximate and chemical composition of experimental animal diets and mean rumen pH values in dry season (DS) and rainy season (RS).

Items	BA ^1^		IM ^2^		CP ^3^		TA ^4^	
	DS	RS	DS	RS	DS	RS	Forage	BR
Proximate composition (% dry matter)								
Dry matter	23.97	24.1	23.87	18.31	23.32	26.12	24.71	39
Organic matter	91.53	90.98	89.45	84.86	91.79	96.88	95.35	94.77
Crude protein	7.86	8.72	7.56	8.86	7.73	28.03	9.38	8.27
NDF	73.23	79.07	68.72	70.91	75.73	55.69	68.96	54.36
NFC	9.07	1.79	11.79	3.09	6.27	5.16	13.85	29.58
ADF	44.99	54.9	40.05	43.9	55.8	22.55	44.51	38.08
Ether extract	1.36	1.4	1.38	1.99	1.64	8	2.06	6.52
TDN	51.48	42.2	56.09	52.5	41.35	72.48	51.93	57.94
Ash	8.47	9.02	10.55	15.14	8.21	3.12	6.17	5.23
Mean rumen pH								
	7.18	7.53	7.23	7.35	7.36	7.13	6.86	

BR = Brewery residue; NDF = Neutral Detergent Fiber; NFC = Non-fibrous carbohydrates; ADF = Acid detergent fiber; TDN = Total digestible nutrients. ^1^ Ecosystem—Baixo Amazonas (BA): In this breeding ecosystem, buffaloes are raised in the traditional way on pasture, in native pastures in areas subject to flooding. During the collection of pastures, there was also an availability of cultivated grasses *Panicum maximum* cv. Mombasa and *Bhachiaria brizantha*. ^2^ Ecosystem—Ilha do Marajó (IM): In this ecosystem, buffaloes are reared in a traditional system, fed exclusively on pasture, with grasses native to areas subject to flooding, such as *Panicum elephantipes*, *Leersia hexandra*, and *Hymenachne amplexicaulis*. ^3^ Ecosystem—Continente do Pará (CP): In this ecosystem, the animals feed on cultivated pastures (*Panicum maximum* cv. Mombaça and *Bhachiaria humidicola*), in areas originally forested (* the animals received wet brewery waste during the dry period). ^4^ Ecosystem—Tomé-Açu (TA): Feed consisted of sorghum silage, soybean meal, wet sorghum premix, and commercial feed (high performance core).

## Data Availability

The data presented in this study are available from the corresponding author upon reasonable request.

## Supplementary Materials

The following supporting information can be downloaded at: https://www.mdpi.com/article/10.3390/ani13243858/s1.
